# Metabolic and lipidomic profiling of steatotic human livers during *ex situ* normothermic machine perfusion guides resuscitation strategies

**DOI:** 10.1371/journal.pone.0228011

**Published:** 2020-01-24

**Authors:** Siavash Raigani, Negin Karimian, Viola Huang, Anna M. Zhang, Irene Beijert, Sharon Geerts, Sonal Nagpal, Ehab O. A. Hafiz, Fermin M. Fontan, Mohamed M. Aburawi, Paria Mahboub, James F. Markmann, Robert J. Porte, Korkut Uygun, Martin Yarmush, Heidi Yeh

**Affiliations:** 1 Division of Transplantation, Department of Surgery, Massachusetts General Hospital, Harvard Medical School, Boston, Massachusetts, United States of America; 2 Center for Engineering in Medicine, Massachusetts General Hospital and Shriners Hospital for Children, Boston, Massachusetts, United States of America; 3 Tufts University School of Medicine, Boston, Massachusetts, United States of America; 4 Section of Hepatobiliary Surgery and Liver Transplantation, Department of Surgery, University of Groningen, University Medical Center Groningen, Groningen, Netherlands; 5 Electron Microscopy Research Division, Theodor Bilharz Research Institute, Giza, Egypt; Medizinische Fakultat der RWTH Aachen, GERMANY

## Abstract

There continues to be a significant shortage of donor livers for transplantation. One impediment is the discard rate of fatty, or steatotic, livers because of their poor post-transplant function. Steatotic livers are prone to significant ischemia-reperfusion injury (IRI) and data regarding how best to improve the quality of steatotic livers is lacking. Herein, we use normothermic (37°C) machine perfusion in combination with metabolic and lipidomic profiling to elucidate deficiencies in metabolic pathways in steatotic livers, and to inform strategies for improving their function. During perfusion, energy cofactors increased in steatotic livers to a similar extent as non-steatotic livers, but there were significant deficits in anti-oxidant capacity, efficient energy utilization, and lipid metabolism. Steatotic livers appeared to oxidize fatty acids at a higher rate but favored ketone body production rather than energy regeneration via the tricyclic acid cycle. As a result, lactate clearance was slower and transaminase levels were higher in steatotic livers. Lipidomic profiling revealed ω-3 polyunsaturated fatty acids increased in non-steatotic livers to a greater extent than in steatotic livers. The novel use of metabolic and lipidomic profiling during *ex situ* normothermic machine perfusion has the potential to guide the resuscitation and rehabilitation of steatotic livers for transplantation.

## Introduction

Liver transplantation remains the only cure for end-stage liver disease. However, there is a significant shortage of donor livers resulting in waitlist mortality rates approaching 20% [1]. One major contributor to the shortage is the high discard rate of steatotic livers [2]. Transplantation of livers with moderate (30–60%) and severe (>60%) macrosteatosis is associated with increased rates of primary non-function, early allograft dysfunction (EAD), and decreased graft survival [3, 4]. As a result, transplantation with such organs is not recommended.

Poor graft function following transplantation of steatotic livers is attributed to multiple physical and metabolic abnormalities, culminating in decreased ability to tolerate ischemia-reperfusion injury (IRI), which occurs during procurement, cold storage, and implantation. At an anatomic level, the large lipid vesicles seen in macrosteatosis compress adjacent sinusoids resulting in reduced sinusoidal perfusion compared to non-steatotic livers [5]. The reduced sinusoidal flow at baseline becomes more pronounced following IRI and can both limit the ability to recover from acute injury and promote further damage [6]. At the metabolic level, steatosis is associated with decreased hepatocyte adenosine triphosphate (ATP) stores at baseline, as well as impaired recovery of ATP stores after depletion [7–9]. Recovery from IRI is an ATP-dependent process [10], indicating that steatotic livers again have decreased likelihood of adequate function following transplantation. Moreover, steatotic hepatocytes have increased sensitivity to oxidative stress due to a chronic reduction in anti-oxidant capacity and increased reactive oxygen species [11], which can lead to graft necrosis following transplantation.

Normothermic machine perfusion (NMP) has already been demonstrated to decrease organ discard rates and EAD, with equivalent graft survival compared to conventional static cold storage (SCS) in spite of worse donor liver characteristics [12]. The ability to use steatotic donor livers for transplantation without compromising patient outcomes would dramatically increase the supply of available livers [13]. Machine perfusion provides an important platform for pharmacologic intervention to rehabilitate these livers that would otherwise be discarded. However, the deficiencies of steatotic livers during NMP at a cellular level are not well defined, as fatty liver disease has many systemic contributors that are hard to separate from intrinsic hepatocyte defects *in vivo* [14].

*Ex situ* NMP also offers the perfect platform to study liver metabolism in isolation. We therefore performed the first reported metabolic and lipidomic profiling of steatotic and non-steatotic human livers during *ex situ* NMP.

## Methods

### Donor livers

8 human donor livers (5 steatotic, 3 non-steatotic), declined for transplantation by all transplant centers, with consent for research between August 2016 and April 2018 were included in this study. All donor livers were received through New England Donor Services (NEDS); no organs were procured from prisoners. Patients or their surrogates (including parents or legal guardians) provided informed consent for use of donor organs in research; all consent was obtained by donation coordinators employed by NEDS. No vulnerable populations were involved in this study. The Massachusetts General Hospital Institutional Review Board (IRB) and the NEDS approved this study (No. 2011P001496), and all studies were carried out in accordance with IRB and NEDS approved guidelines. The main reasons for organ decline were: 1) more than 30% macrosteatosis of the donor livers evaluated by histology at the time of procurement, and 2) the combination of donation after circulatory death (DCD) and old donor age or prolonged ischemia time (S1 Table).

### Procurement and preparation of liver grafts

All donor livers were procured based on the standard technique of *in situ* cold flush using University of Wisconsin (UW) preservation solution. Procurement techniques for donation after brain death (DBD) or donation after circulatory death are previously described [15]. Warm ischemic time (WIT) is defined as time from circulatory arrest to *in situ* cold flushing for DCD livers. Cold ischemic time (CIT) is defined from *in situ* cold flushing to start of machine perfusion.

Upon arrival to our perfusion lab, back table preparation of the livers was performed as described previously [16].

### Machine perfusion

Liver grafts were perfused for three hours using a pressure and temperature-controlled perfusion device, Liver Assist (Organ Assist, Groningen, Netherlands), using a previously established protocol [16]. During NMP, livers were perfused with a portal pressure of 7 mmHg and mean hepatic arterial pressure of 70 mmHg. The temperature of the perfusate was maintained between 36–38°C. The perfusate consisted of Williams’ medium E supplemented with hydrocortisone, insulin, heparin [15], albumin, fresh frozen plasma and a hemoglobin-based oxygen carrier, Hemopure® (HbO_2_ Therapeutics LLC, Souderton, PA, USA). Detailed composition is provided in the S1 Methods. The choice of oxygen carrier was based on a study demonstrating the efficacy of Hemopure compared to packed red blood cells during NMP of human liver grafts [17, 18]. Perfusate samples were collected from the arterial inflow and venous outflow at 30-minute intervals. Blood gas and chemistry analysis was performed using i-STAT Blood Analyzer (Abbott Point of Care Inc., Princeton, NJ, USA).

### Evaluation of hepatic injury and function

Perfusion dynamics were recorded every 30 minutes. Vascular resistance was defined as perfusion pressure divided by flow rate (mmHg^1^ min^1^ mL^-1^). Alanine aminotransferase (ALT) was determined using Piccolo Xpress Chemistry Analyzer (Abbott Point of Care Inc., Princeton, NJ, USA). The volume of bile produced was recorded at 1-hour intervals. Two wedge liver biopsies were collected immediately prior to perfusion and then hourly during perfusion. One tissue sample was snap-frozen in liquid nitrogen for future analysis or and the other preserved in 10% formalin for histology. Perfusate nitric oxide levels were measured using a colorimetric assay (Biovision, #K262).

### Histological assessment

After formalin fixation, tissue samples were paraffin-embedded, and stained with hematoxylin-eosin (H&E). Tissue slides were evaluated for macrosteatosis content and preservation injury by a blinded expert pathologist [19]. Livers with ≥30% macrosteatosis on pre-perfusion biopsy were allocated to the “steatotic” group.

### Targeted metabolomics analysis–energy cofactors

Frozen liver biopsies from each time point (~25mg) were pulverized in liquid nitrogen and analyzed for metabolic cofactors using a targeted multiple reaction monitoring analysis on a Sciex TripleTOF 6600 Quadruple Time-Of-Flight system, performed at the principle research institution. Metabolites were extracted using the protocol provided by Yuan *et al*. [20]. Concentrations of hepatic adenosine tri-, di-, and mono- phosphate (ATP, ADP, AMP), and reduced and oxidized nicotinamide dinucleotide phosphate (NADPH, NADP^+^) were quantified. Energy charge was calculated as: [ATP + ADP*0.5] / [ATP+ADP+AMP].

### Untargeted metabolomic and lipidomic analysis

Tissue biopsies of three livers from each group were analyzed for 1600 compounds of known identity by Metabolon, Inc. (Durham, North Carolina). Metabolite analysis utilized a Waters ACQUITY ultra-performance liquid chromatography (UPLC) and a Thermo Scientific Q-Exactive high resolution/accurate mass spectrometer interfaced with a heated electrospray ionization (HESI-II) source and Orbitrap mass analyzer operated at 35,000 mass resolution. Principal component analysis (S1 Fig), detailed methods, and statistical approach are provided in the S1 Methods. The complete metabolomic and lipidomic profile for both groups is provided in the S1 Dataset.

### Statistical analysis

Demographic and perfusion data are presented as the mean ± standard error of the mean (SEM), unless otherwise specified, with statistical significance defined as P<0.05. Wilcoxon’s rank-sum (Mann-Whitney U) test and Fischer’s exact test were used for continuous and categorical comparisons, respectively. Repeated measures data were analyzed using a random intercept mixed model with a categorical effect of time. If between-group comparisons were made, the categorical effect of group and the group by time interactions were added to the model. Statistical analysis was performed using Stata 15.1 (StataCorp, College Station, Texas) and SPSS (IBM, Armonk, New York). Graphics were created using Prism 8 (GraphPad, San Diego, California).

## Results

### Liver characteristics and hemodynamics

**Fig 1:**
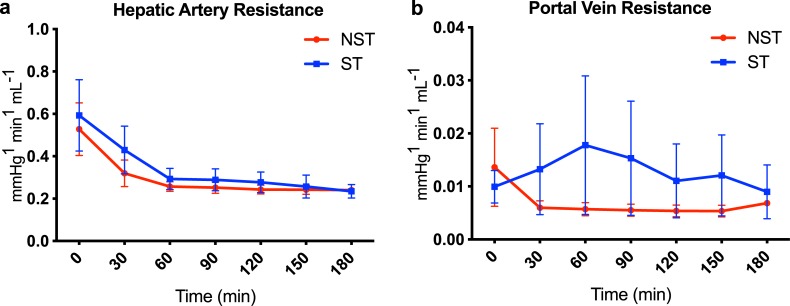
Vascular resistance during perfusion of steatotic and non-steatotic livers. Steatotic livers tend to have higher vascular resistance at initiation of perfusion, which improves with time. No significant differences were observed between the groups with mixed model analysis. (a) Hepatic artery resistance and (b) Portal vein resistance are shown during 3 hours of normothermic machine perfusion. Data shown as mean ± SEM. ST, steatotic; NST, non-steatotic.

A comparison of donor characteristics among the steatotic (ST) and non-steatotic (NST) livers is provided in Table 1. ST livers tended to have higher hepatic arterial and portal venous resistance, but differences were not statistically significant (Fig 1).

**Table 1:** Baseline donor characteristics and ischemic times of steatotic and non-steatotic livers.

Variables	Non-steatotic	Steatotic
	*(n = 3)*	*(n = 5)*
**Age (years)**	44 (28–60)	48.4 (37–55)
**Gender (male)**	2 (66%)	4 (80%)
**BMI (kg/m**^**2**^**)**	24.7 (16.9–32.5)	32.6 (25.8–37.9)
**Type of donor liver**		
DCD	2 (66%)	2 (40%)
DBD	1 (34%)	3 (60%)
**WIT (min)**	34*	27*
**CIT (min)**	660 (360–930)	735 (463–935)
**Weight of liver (g)**	1617 (1300–2200)	2302 (2011–2830)

Estimates provided are the mean with range in parentheses.

* Indicates only one available data point for variable.

### Histological changes during perfusion

**Fig 2:**
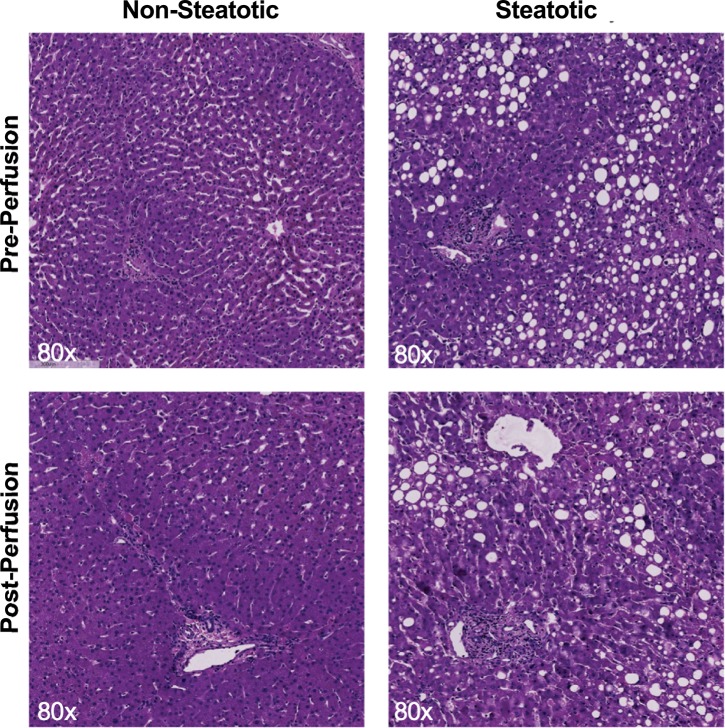
Histologic differences between steatotic and non-steatotic livers before and after perfusion. Representative H&E-stained liver sections from steatotic and non-steatotic livers shown pre-perfusion and post-perfusion (3 hours of normothermic machine perfusion). Steatotic liver biopsies demonstrate large lipid macrodroplets in the cytoplasm of hepatocytes. No significant change in macrosteatosis content was observed after three hours of perfusion in steatotic and non-steatotic livers.

Liver biopsies collected before and after perfusion were evaluated for percent macrosteatosis. Percent macrosteatosis did not change significantly in either group after 180 minutes of NMP (Table 2). However, ST livers demonstrated severe sinusoidal congestion, disruption of the central venous endothelial lining, and hepatocyte swelling compared to NST livers (Fig 2).

**Table 2:** Histologic macrosteatosis of perfused livers.

		Macrosteatosis Percentage	
Liver #	Group	Pre-Perfusion	Post-Perfusion
1	Non-steatotic	<5%	<5%
2	Non-steatotic	<5%	<5%
3	Non-steatotic	15%	10%
1	Steatotic	30%	30%
2	Steatotic	60%	60%
3	Steatotic	30%	30%
4	Steatotic	30%	30%
5	Steatotic	80%	80%

Based on wedge liver biopsies taken prior to initiation of perfusion (pre-perfusion) and after 3 hours of perfusion (post-perfusion)

### Liver function

**Fig 3:**
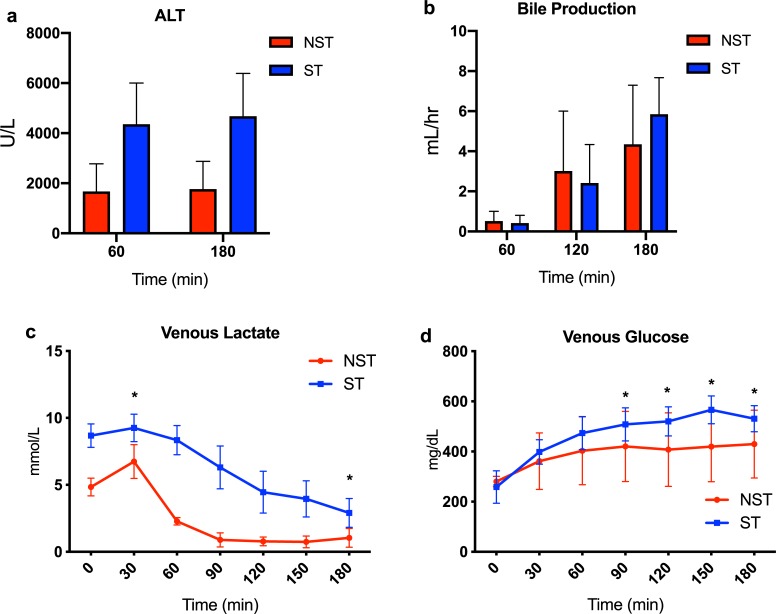
Characterization of liver function and injury during perfusion. (a) Release of alanine aminotransferase. ALT was higher in steatotic livers but did not reach significance. (b) Bile production measured at each hour of perfusion. Bile production increased at comparable volumes between groups. (c) Lactate content measured in the venous outflow of livers during perfusion. ST livers have higher lactate content and slower clearance compared to NST livers. (d) Glucose content measured in the venous outflow. ST livers release higher levels of glucose during perfusion compared to NST livers. * indicates P<0.05 for random intercept mixed model comparison of repeated measures data between groups. Comparisons are made at each time point between groups with respect to the measured change from time = 0 minutes. Data shown as mean ± SEM. ST, steatotic; NST, non-steatotic.

ALT levels were higher in the ST compared to NST livers (Fig 3A), although the difference was not statistically significant (4672 vs. 1757 U/L at 180 min, P = 0.18). Hourly bile production was also similar in both groups (Fig 3B). Venous lactate levels were higher in the ST livers throughout perfusion. Mixed model analysis demonstrated a significant difference in the rate of change in venous lactate between the two groups from initiation of perfusion to 30 and 180 minutes (Fig 3C). Glucose concentrations in the perfusate increased in both groups and were significantly higher in the ST livers after 90 minutes of perfusion (Fig 3D).

### Oxidative stress

**Fig 4:**
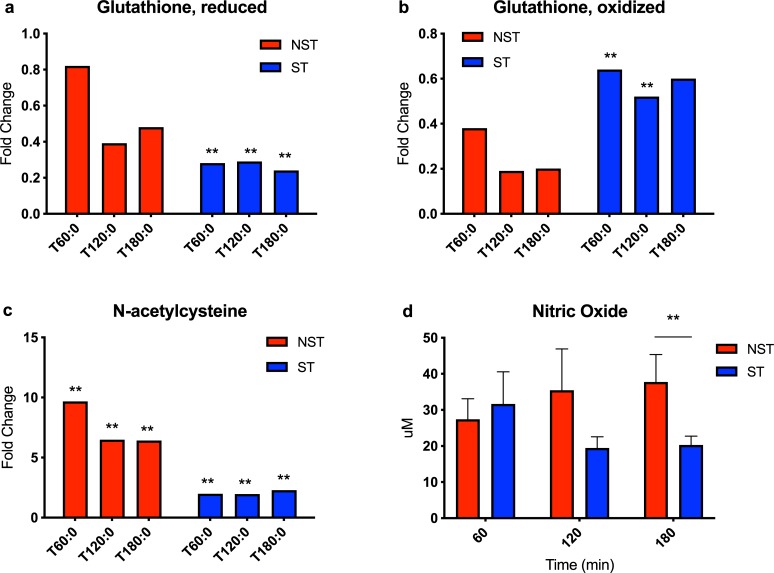
Redox factor changes in steatotic and non-steatotic livers during perfusion. Ratio of metabolite concentration fold change at each hour of perfusion compared to pre-perfusion concentration shown for (a) reduced glutathione, (b) oxidized glutathione, and (c) N-acetylcysteine. (d) Concentration of nitric oxide in perfusate samples. Steatotic livers demonstrate significant deficits in redox capacity and NO synthesis during perfusion. ** indicates P≤0.05, * indicates 0.05<P<0.10. ST, steatotic; NST, non-steatotic; x-axis represents fold change at 60, 120, and 180 minutes compared to pre-perfusion concentrations for (a-c).

Several antioxidant metabolites were closely examined. Glutathione is the major intracellular redox agent and the ratios of its reduced and oxidized form indicate the degree of cellular oxidative stress [21]. Reduced glutathione levels decreased hourly in ST livers (0.24–0.29 fold change, P<0.05 for each hour, Fig 4A), and did so to a much lesser extent in NST livers (range 0.39–0.82, P not significant for each hour). Oxidized glutathione levels decreased in both groups, with ST livers decreasing less from baseline compared to NST livers (Fig 4B).

N-acetylcysteine (N-Ac) is an effective free radical scavenger and an important precursor to glutathione synthesis by providing the L-cysteine substrate [22]. N-Ac levels in NST livers increased much more during perfusion (range 6.41–9.65 fold change, P<0.05 for each hour), than in ST livers (range 1.94–2.27 fold, P<0.05 for each hour, Fig 4C).

Reactive oxygen species (ROS) suppress levels of nitric oxide (NO), a vasoprotective endothelial signaling molecule, both by scavenging and by decreasing production [23]. NO perfusate levels increased at each hour in NST livers and were significantly higher at 180 minutes perfusion compared to ST livers (37.7 vs. 20.2 μM, P = 0.0253, Fig 4D).

### ATP production

**Fig 5:**
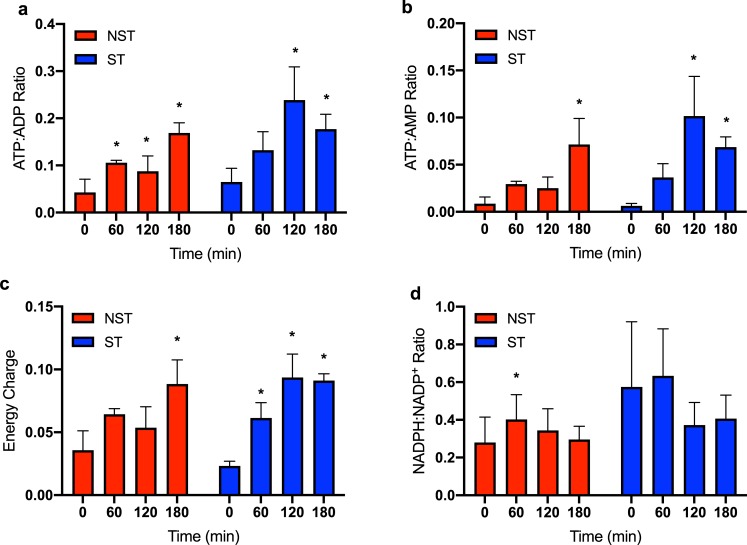
Analysis of energy cofactor changes in steatotic and non-steatotic liver tissue during perfusion. (a) Measured ATP:ADP ratio, (b) ATP:AMP ratio, (c) energy charge, and (d) NADPH:NADP^+^ ratios at each hour of perfusion. Energy charge was calculated as (2*ATP + ADP)/(ATP + ADP + AMP). Within group comparisons at 60, 120, and 180 minutes are made to a pre-perfusion measurement (time = 0 min). * indicates P<0.05 for random intercept mixed model comparison of repeat measures data to pre-perfusion levels. Data shown are mean ± SEM. ST, steatotic; NST, non-steatotic.

ATP:ADP and ATP:AMP ratios were significantly higher at end-perfusion compared to pre-perfusion in both groups (Fig 5A and 5B). Energy charge was not significantly increased until 180 minutes of perfusion in NST livers, whereas it was significantly increased by 60 minutes perfusion in ST livers (Fig 5C). No significant changes were seen in NADPH:NADP^+^ ratios except at 60 minutes in the NST livers (Fig 5D). Comparison of cofactor ratios between NST and ST groups at each time point did not demonstrate any significant differences.

#### Fatty acid oxidation

**Fig 6:**
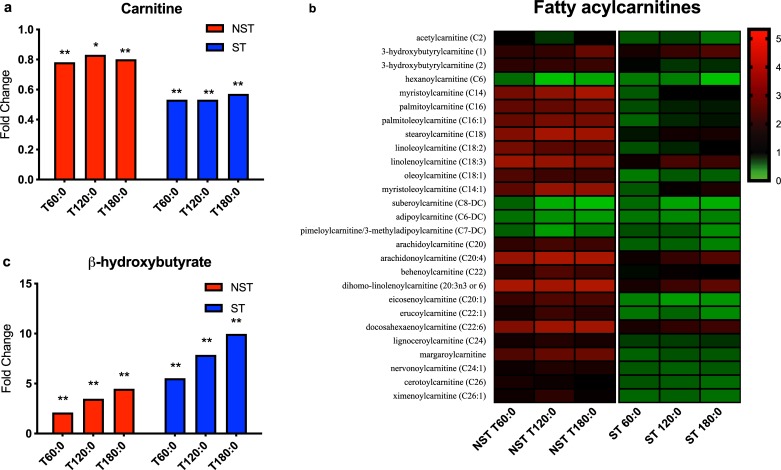
Metabolic capacity of fatty acid oxidation in steatotic and non-steatotic livers during perfusion. (a) Carnitine and (b) β-hydroxybutyrate concentration fold change at each hour of perfusion compared to pre-perfusion concentration. (c) Heatmap of fatty acylcarnitine metabolite concentration fold change at each hour of perfusion. Steatotic livers demonstrate upregulation of fatty acid oxidation, with resultant depletion of carnitine and fatty acylcarnitine levels during perfusion, but favor greater ketone synthesis compared to non-steatotic livers. ** indicates P≤0.05, * indicates 0.05<P<0.10. ST, steatotic; NST, non-steatotic; x-axis represents fold change at 60, 120, and 180 minutes compared to pre-perfusion concentrations.

Carnitine is an essential cofactor in the transport of fatty acids into mitochondria for oxidation. Carnitine levels decrease in both groups, though to a larger degree in ST livers (ST range 0.53–0.57 fold change vs. NST range 0.78–0.83, P<0.10 for all). However, fatty acylcarnitine levels increased over time in NST livers and decreased in ST livers (Fig 6A and 6B).

Fatty acid oxidation (FAO) produces acetyl CoA, the necessary precursor for the tricyclic acid cycle. Ketones bodies such as acetoacetate and β-hydroxybutyrate are synthesized from acetyl CoA in the liver when glycogen stores are depleted as an alternative energy source for the brain, heart, and muscle. Interestingly, β-hydroxybutyrate levels increased more in ST livers (range 5.51–9.94 fold change, P<0.05) than NST livers (range 2.08–4.64 fold, P<0.05, Fig 6C).

#### Tricyclic acid cycle

**Fig 7:**
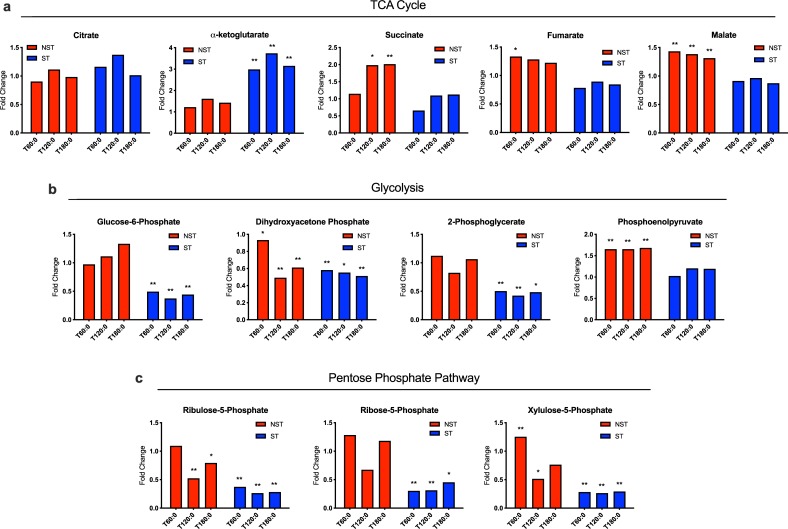
Metabolic analysis of cellular metabolism in steatotic and non-steatotic livers during perfusion. Ratio of metabolite concentration fold change at each hour of perfusion compared to pre-perfusion concentration shown for (a) tricyclic acid cycle, (b) glycolysis, and (c) pentose phosphate pathway. Steatotic livers demonstrate decreased flux through each metabolic pathway. ** indicates P≤0.05, * indicates 0.05<P<0.10. ST, steatotic; NST, non-steatotic; x-axis represents fold change at 60, 120, and 180 minutes compared to pre-perfusion concentrations.

The tricyclic acid cycle consumes acetyl CoA to generate reduced nicotinamide adenine nucleotide (NADH), which is necessary to regenerate ATP in oxidative phosphorylation. There is significant accumulation of α-ketoglutarate (range 2.98–3.14 fold, P<0.05) in ST livers, an upstream metabolite in the tricyclic acid (TCA) cycle. Conversely, NST livers preferentially accumulate downstream metabolites, such as succinate, fumarate, and malate (Fig 7A).

#### Glucose metabolism

Glycolysis is the breakdown of glucose to produce 2 molecules of pyruvate for use in the TCA cycle. The pentose phosphate pathway runs in parallel to glycolysis and generates NADPH and ribose-5-phosphate, a precursor to nucleotide synthesis. Tissue levels of glycolytic intermediates glucose-6-phosphate (G6P), dihydroxyacetone phosphate (DHAP), 2-phosphoglycerate (2-PG), and phosphoenolpyruvate (PEP) are lower during perfusion in ST livers compared to NST livers (Fig 7B). Metabolites in the pentose phosphate pathway similarly decreased more in ST livers compared to NST livers (Fig 7C).

### Lipidomic profile

As expected, NST livers have different lipid composition compared to ST livers (S2 Table). NST livers contain a much smaller proportion of triacylglycerol (TAG) content (37.1% pre-perfusion and 34.9% after NMP), than ST livers (61.6% pre-perfusion and 62.9% after NMP). The triacylglycerol:diacylglycerol (TAG:DAG) ratio decreased during perfusion in both groups, but to a much larger degree in the NST group (NST -34% change vs. -10% change, P = 0.0495).

**Fig 8:**
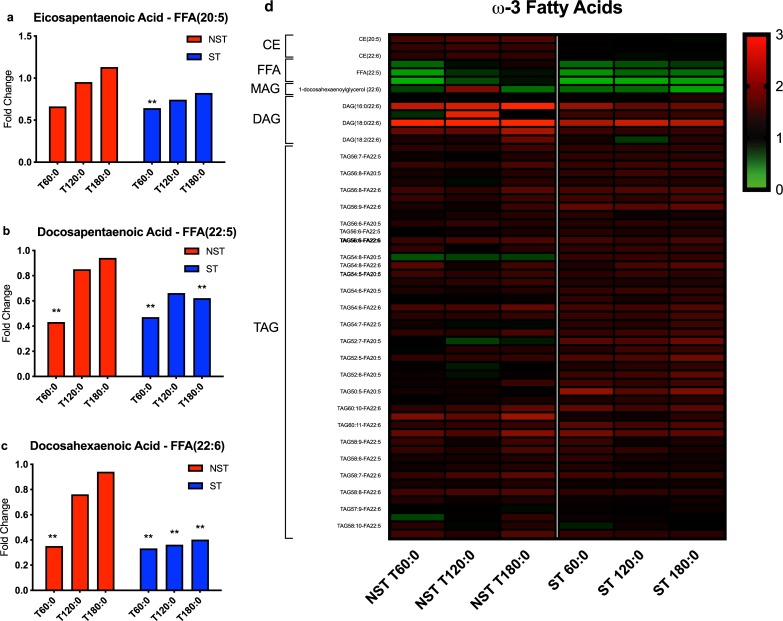
ω-3 fatty acid lipidomic profiles of steatotic and non-steatotic livers during perfusion. Ratio of metabolite concentration fold change at each hour of perfusion compared to pre-perfusion concentration shown for the free fatty acid (FFA) form of (a) eicosapentaenoic acid (EPA), (b) docosapentaenoic acid (DPA), and (c) docosahexaenoic acid (DHA). (d) Heatmap of EPA, DPA, and DHA concentration fold changes in cholesterol ester (CE), FFA, monoacylglycerol (MAG), diacylglycerol (DAG), and triacylglycerol (TAG) forms. ** indicates P≤0.05, * indicates 0.05<P<0.10. ST, steatotic; NST, non-steatotic; x-axis represents fold change at 60, 120, and 180 minutes compared to pre-perfusion concentrations.

The omega-3 (ω-3) polyunsaturated fatty acids (PUFA), eicosapentaenoic acid (EPA, 20:5n-3) and docosahexaenoic acid (DHA, 22:6n-3), as well as the intermediary docosapentaenoic acid (DPA, 22:5n-3), decreased initially in their free fatty acid (FFA) form, returning to baseline (fold change ratios approaching 1.0) at end-perfusion in NST livers but remaining low in ST livers (Fig 8A–8C). As perfusion time increased, ST livers appeared to have higher ω-3 PUFA levels in TAG form and lower levels in DAG form compared to NST livers. In cholesterol ester form (CE), EPA, DHA, and DPA levels increased significantly in NST livers during perfusion but did not change in ST livers (Fig 8D).

Arachidonic acid (AA, 20:4n-6), an omega-6 (ω-6) PUFA increased in both groups in FFA form, though to a larger extent in NST livers. AA levels in DAG form increased in NST livers during perfusion, but no similar pattern was observed in the ST livers. In TAG form, AA increased slightly overall in both NST and ST livers (S2 Fig).

### Bile acids

**Fig 9:**
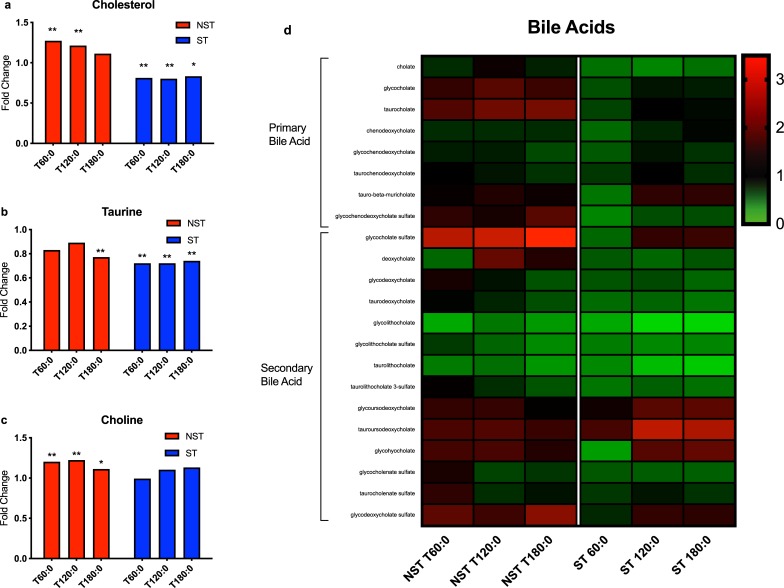
Bile acid profiles of steatotic and non-steatotic livers during perfusion. Ratio of metabolite concentration fold change at each hour of perfusion compared to pre-perfusion concentration shown for (a) cholesterol, (b) taurine, and (c) choline. (d) Heatmap of primary and secondary bile acid concentration fold changes during perfusion. ** indicates P≤0.05, * indicates 0.05<P<0.10. ST, steatotic; NST, non-steatotic; x-axis represents fold change at 60, 120, and 180 minutes compared to pre-perfusion concentrations.

Cholesterol, taurine, and choline are substrates for bile acid synthesis in the liver. Tissue cholesterol increased during the first 2 hours in NST livers, but decreased in ST livers (P<0.05 for all, Fig 9A). Taurine levels decreased throughout perfusion in ST livers (P<0.05 for all), but only at the end of perfusion in NST livers (Fig 9B). Choline levels increased significantly in NST livers but not in ST livers (Fig 9C). Primary and secondary bile acids largely decreased in ST livers compared to NST livers. Two exceptions to this pattern include glycoursodeoxycholate and tauroursodeoxycholate, which increased in ST livers during perfusion (Fig 9D).

## Discussion

Resuscitating steatotic livers to expand the donor liver pool will be enhanced by knowledge of the metabolic deficiencies specific to these organs. This data may also inform therapies directed at treating hepatic steatosis *in vivo*. This study is the first of its kind to examine the metabolic and lipidomic profiles of steatotic livers during oxygenated normothermic machine perfusion. During NMP, steatotic livers suffer higher levels of oxidative stress, deficiencies in cellular metabolism, and altered lipid and bile acid metabolism compared to non-steatotic livers.

Additionally, this work unveiled deficiencies in steatotic livers that may hinder optimal function, while indicating potential solutions. For example, we found that ST livers generate less NO than NST livers, which in the post-transplant setting would impair microcirculation and increase endothelial damage. This could be the result of deformed sinusoids or uncoupling of nitric oxide synthase leading to decreased NO availability as a result of increased ROS [5, 24, 25]. Indeed, chronic oxidative stress and inflammation from excessive FFA uptake and FAO is a hallmark of NAFLD and progression to non-alcoholic steatohepatitis (NASH) [26]. We confirmed that this is an intrinsic state of ST livers and not solely a reaction to the systemic environment of metabolic syndrome by finding that ST livers during NMP still rapidly deplete their supply of reduced glutathione and N-Ac. Perfusate supplementation with anti-oxidants, such as Vitamin E, N-Ac, or L-alanyl-glutamine, could counteract the acute oxidative stress and ROS that accompany IRI [27–30].

We also report the novel finding of fatty acylcarnitine metabolism during NMP, which increased in concentration during perfusion of NST livers but decreased in ST livers. Transport of fatty acylcarnitines into the mitochondria is an essential step in FAO. Carnitine supplementation in human steatotic liver perfusion will likely decrease macrosteatosis by increasing FAO, and has been shown to be effective in a rat fatty liver perfusion model [31, 32]. Interestingly, in patients with NAFLD and NASH, absolute acylcarnitine levels actually increase with disease progression, but this may be an attempt at mobilizing lipids for oxidation while still being insufficient for the degree of steatosis [33], as suggested by our *ex situ* NMP results. Given the decreasing TAG:DAG ratio and increasing acylcarnitine concentrations, NST livers appear to mobilize TAG stores much more efficiently for mitochondrial FAO. Conversely, ST livers quickly deplete the available fatty acylcarnitine pool and are less capable of mobilizing TAG stores for continued energy production.

Furthermore, NST livers favor efficient energy utilization pathways, as demonstrated by comparatively lower ketone concentration increases and higher downstream TCA cycle metabolite ratios. In comparison, ST livers demonstrate larger increases in ketone concentrations ratios and higher α-ketoglutarate ratios, indicating the energy potential from FAO is largely dissipated through excessive ketone body synthesis. This occurs in spite of higher perfusate glucose levels and insulin supplementation, which should decrease feedback to the liver that the hypothetical brain and muscle bodies require ketones as an alternative energy source. Elevated perfusate glucose concentrations in ST livers is also consistent with insulin-resistant profiles seen in patients with non-alcoholic fatty liver disease (NAFLD) [14].

Interestingly, ST and NST livers regenerated similar ATP and energy charge ratios despite abundant evidence of metabolic deficiencies. This is the first study to demonstrate that steatotic and non-steatotic livers are able to regenerate ATP and energy charge during NMP, which is consistent with prior evidence from subnormothermic perfusion studies [15, 34]. The perfusion data presented here support the idea that NMP partially resuscitates steatotic livers, improving ATP and energy charge. Lee *et al*. demonstrated increased degradation of oxidative phosphorylation subunits in a proteomic study of diet-induced NAFLD in mice [35]. The resulting reduced efficiency of oxidative phosphorylation, the pathway downstream to the TCA cycle, as a result of this supports the evidence in our study demonstrating a metabolic barrier in the TCA cycle seen in ST livers. The equivalent ATP ratios between ST and NST livers therefore may indicate an invisible ceiling to energy production in ST livers rather than a marker of adequate resuscitation. All perfused liver except one ST liver met the viability criteria for transplant of Laing et al. [36], though ST livers required longer perfusion time to meet criteria and met fewer total criteria compared to NST livers (S3 Table).

Another unique finding in this study is the pattern of ω-3 PUFA metabolism during perfusion. DHA and EPA concentration ratios decrease in FFA form during perfusion in ST livers and do not return to pre-perfusion levels, as they do in NST livers. Furthermore, DHA and EPA were more favorably stored in TAGs in ST livers, whereas NST livers favored the mobilized DAG, FFA, and CE forms. The downstream products of EPA and DHA include resolvins, protectins, and maresins, generated via cyclooxygenase and lipoxygenase pathways [37]. These metabolites possess anti-inflammatory and inflammation-resolving abilities, and have been shown to inhibit tumor necrosis factor-α (TNFα) and interleukin-1β (IL-1β) production [38–40]. DHA has also been demonstrated to activate peroxisome proliferator-activated receptor-γ (PPAR-γ), which is an inhibitor of the proinflammatory nuclear receptor, NF-κB [37]. Preliminary data from our lab indicates that ST livers express more inflammatory cytokines than NST livers (manuscript in preparation); thus, perfusion supplementation with EPA and DHA, currently unexplored, could improve *ex situ* resuscitation of steatotic livers by favoring anti-inflammatory and inflammation-resolving pathways. In longer term *in vivo* studies in humans, as well as in mice, ω-3 PUFA supplementation has been demonstrated to improve cardiovascular risk, reduce steatosis, and decrease hepatic IRI [41–45].

This study is also the first to demonstrate how ST and NST livers modulate bile acid synthesis during NMP. Given relative deficiencies in cholesterol, choline, and taurine concentrations with increasing perfusion time, ST livers have similar overall deficits in primary and secondary bile acid concentrations. Bile acid activation of the farnesoid X receptor (FXR) is a major signaling pathway in hepatocytes. FXR activation has been demonstrated to increase FAO through PPAR-α activation, increase insulin sensitivity, and modulate triglyceride and lipoprotein metabolism [46]. In a randomized prospective clinical trial, treatment of patients with non-cirrhotic NASH using the semi-synthetic FXR agonist, obeticholic acid, demonstrated significant improvement in fibrosis, steatosis, hepatocellular ballooning, and lobular inflammation compared to placebo [47]. By extension, perfusate supplementation with a natural or synthetic FXR agonist could similarly optimize a steatotic liver’s cellular metabolism during *ex situ* perfusion.

One limitation of this study was the relatively small group sizes. Research using discarded human livers lends itself to significant heterogeneity between donors and randomization is not feasible, so there is significant variance in not only demographic data, but also metabolic variability based on a donor’s medical history, procurement technique, and ischemic times. As a result, this study was underpowered to compare perfusion dynamics between groups. However, corroborating prior studies, we did find that ST livers cleared lactate at a slower rate and had higher hepatic arterial resistance during NMP [34, 48]. Notably, the primary endpoint of this study was to compare the metabolic and lipidomic profiles of steatotic and non-steatotic livers and not their perfusion characteristics, which have been extensively described in prior studies [48, 49]. Furthermore, the use of multiple comparison corrections and false-discovery rates, employed in the untargeted metabolic and lipidomic profiling, can compensate for the small group sizes. This study also used Hemopure, a synthetic hemoglobin-based oxygen carrier, rather than traditionally used packed red blood cells (PRBC). We felt this provided a more consistent representation of the metabolic changes occurring during perfusion, as PRBC for research use are often obtained from multiple donors and could further confound the metabolite data in the setting of erythrocyte hemolysis and immune-mediated phenomena [18]. While we expect the use of a synthetic carrier helps minimize experimental variability, some metabolic differences would be expected if a PRBC-based perfusate is used in future studies, and comparisons with literature data should be done with caution accordingly. Similarly, experience with use of Hemopure as the primary oxygen carrier during perfusion is expanding and has several benefits, including a long shelf-life, low inflammatory profile, and non-thrombogenicity [17, 18, 50]. Finally, we chose a three-hour perfusion as this is generally the time frame in which transplant surgeons determine viability [36], as studies have noted that a liver appears to reach a steady state in terms of perfusion parameters, functional biomarkers, liver injury, and energy status by the end of three hours [15, 49]. Defining the metabolic deficits during this time seemed to be most productive in identifying strategies to optimize steatotic grafts for transplantation.

The lack of widely accepted criteria for determining when or if a machine-perfused liver is adequately resuscitated for implantation is still a major impediment to broader clinical use of discarded human livers after *ex situ* perfusion, though criteria have been proposed [36]. Real-time lipidomic analysis using rapid evaporation ionization mass spectrometry (REIMS) has been applied to endoscopic colonic polypectomy, as well as oncologic breast and ovarian surgery [51–53]. Research on multiple–omics level data integration is also ongoing [54]. With the development of these rapid analysis techniques, our novel combination of NMP with metabolomic and lipidomic analysis could be integrated into the decision-making process of transplant surgeons.

In conclusion, steatotic livers undergoing oxygenated normothermic machine perfusion have significantly altered metabolic and lipidomic profiles compared to non-steatotic livers. Improving the cellular metabolism of steatotic livers by targeting specific pathways with metabolite supplementation and pharmaceuticals may allow significant resuscitation and rehabilitation of this subset of organs that would otherwise be discarded in the sequence of organ procurement, thereby increasing the supply of organs for transplantation.

## Supporting information

S1 Dataset Metabolomic dataset. Complete metabolomic and lipidomic dataset obtained from untargeted mass spectrometry analysis provided in Excel spreadsheet format.
(XLSX)

S1 Fig Multivariate analysis of metabolomic and lipidomic perfusion profiles. Principal component analysis of ST (blue) and NST (red) livers based on tissue biopsies taken pre-perfusion (filled circle), 60 min (open circle), 120 min (filled diamond), and 180 min (open diamond). (a) 3-dimensional representation of untargeted metabolomic profile demonstrates group-wise clustering of ST and NST during perfusion, whereas pre-perfusion profiles of the two groups are more similar. (b) 2-dimensial representation of untargeted lipidomic profile demonstrates group-wise clustering of ST and NST livers at all time points. ST, steatotic; NST, non-steatotic; PC, principal component.
(EPS)

S2 Fig Arachidonic acid lipid composition during perfusion. Heatmap of arachidonic acid concentration fold changes in cholesterol ester (CE), free fatty acid (FFA), monoacylglycerol (MAG), diacylglycerol (DAG), and triacylglycerol (TAG) forms. ST, steatotic; NST, non-steatotic; x-axis represents fold change at 60, 120, and 180 minutes compared to pre-perfusion concentrations.
(EPS)

S1 Methods Supplementary methods. Provides supplemental methods referenced in main manuscript.
(DOCX)

S1 Table Cause of death for donor livers. Listed cause of death for organ donors whose discarded liver was included in this study.
(DOCX)

S2 Table Lipid composition of perfused livers. Based on wedge liver biopsies taken prior to initiation of perfusion (pre-perfusion) and after 180 minutes of perfusion (post-perfusion). Cholesterol ester (CE), ceramide (CER), diacylglycerol (DAG), dihydroceramide (DCER), free fatty acid (FFA), hexosylceramide (HCER), lactosylceramide (LCER), lysophosphatidylcholine (LPC), lysophosphatidylethanolamine (LPE), phosphatidylcholine (PC), phosphatidylethanolamine (PE), phosphatidylinositol (PI), sphingomyelin (SM), triacylglycerol (TAG).
(DOCX)

S3 Table Viability assessment of perfused livers. Based on viability criteria reported by Laing et al. (reference 35). A perfused liver is deemed viable if it meets 2 or more criteria. NST, non-steatotic liver; ST, steatotic liver; HA, hepatic artery; PV, portal vein.
(DOCX)
