# Supplementary material for: Metabolic and lipidomic profiling of steatotic human livers during ex situ normothermic machine perfusion guides resuscitation strategies
Source: PLoS One. 2020 Jan 24;15(1):e0228011. doi: 10.1371/journal.pone.0228011 (PMC6980574; doi:10.1371/journal.pone.0228011)
Supplement: S1 Table — Listed cause of death for organ donors whose discarded liver was included in this study. (DOCX) [file pone.0228011.s005.docx]

**S1 Table. Cause of death for donor livers.**

|  | Liver # | Cause of death |
| --- | --- | --- |
| Non-steatotic livers | 1  2  3 | Anoxia  Unknown  Anoxia |
| Steatotic Livers | 1  2  3  4  5 | Cerebrovascular accident (stroke)  Cerebrovascular accident (stroke)  Cerebrovascular accident (stroke)  Anoxia  Anoxia |
