# Supplementary material for: Metabolic and lipidomic profiling of steatotic human livers during ex situ normothermic machine perfusion guides resuscitation strategies
Source: PLoS One. 2020 Jan 24;15(1):e0228011. doi: 10.1371/journal.pone.0228011 (PMC6980574; doi:10.1371/journal.pone.0228011)
Supplement: S2 Table — Based on wedge liver biopsies taken prior to initiation of perfusion (pre-perfusion) and after 180 minutes of perfusion (post-perfusion). Cholesterol ester (CE), ceramide (CER), diacylglycerol (DAG), dihydroceramide (DCER), free fatty acid (FFA), hexosylceramide (HCER), lactosylceramide (LCER), lysophosphatidylcholine (LPC), lysophosphatidylethanolamine (LPE), phosphatidylcholine (PC), phosphatidylethanolamine (PE), phosphatidylinositol (PI), sphingomyelin (SM), triacylglycerol (TAG). (DOCX) [file pone.0228011.s006.docx]

**S2 Table. Lipid composition of perfused livers (molar percentage).**

|  | **Non-Steatotic** | | **Steatotic** | |
| --- | --- | --- | --- | --- |
| **Lipid** | Pre | Post | Pre | Post |
| **CE** | 2.04 | 2.26 | 2.40 | 2.16 |
| **CER** | 0.14 | 0.34 | 0.09 | 0.18 |
| **DAG** | 0.80 | 1.13 | 0.91 | 1.04 |
| **DCER** | 0.03 | 0.06 | 0.02 | 0.04 |
| **FFA** | 2.38 | 2.51 | 1.41 | 1.22 |
| **HCER** | 0.04 | 0.05 | 0.02 | 0.02 |
| **LCER** | 0.11 | 0.11 | 0.06 | 0.05 |
| **LPC** | 0.22 | 0.10 | 0.19 | 0.13 |
| **LPE** | 0.11 | 0.07 | 0.11 | 0.08 |
| **PC** | 27.02 | 28.12 | 16.15 | 15.52 |
| **PE** | 25.14 | 25.64 | 14.40 | 14.28 |
| **PI** | 0.42 | 0.47 | 0.21 | 0.24 |
| **SM** | 4.48 | 4.23 | 2.46 | 2.15 |
| **TAG** | 37.07 | 34.93 | 61.57 | 62.89 |

^Based on wedge liver biopsies taken prior to initiation of perfusion (pre-perfusion) and after 180 minutes of perfusion (post-perfusion). Cholesterol ester (CE), ceramide (CER), diacylglycerol (DAG), dihydroceramide (DCER), free fatty acid (FFA), hexosylceramide (HCER), lactosylceramide (LCER), lysophosphatidylcholine (LPC), lysophosphatidylethanolamine (LPE), phosphatidylcholine (PC), phosphatidylethanolamine (PE), phosphatidylinositol (PI), sphingomyelin (SM), triacylglycerol (TAG). Numbers presented are mean molar percentage.^
