# Supplementary material for: Metabolic and lipidomic profiling of steatotic human livers during ex situ normothermic machine perfusion guides resuscitation strategies
Source: PLoS One. 2020 Jan 24;15(1):e0228011. doi: 10.1371/journal.pone.0228011 (PMC6980574; doi:10.1371/journal.pone.0228011)
Supplement: S3 Table — Based on viability criteria reported by Laing et al. (reference 35). A perfused liver is deemed viable if it meets 2 or more criteria. NST, non-steatotic liver; ST, steatotic liver; HA, hepatic artery; PV, portal vein. (DOCX) [file pone.0228011.s007.docx]

**S3 Table. Viability assessment of perfused livers.**

| **Liver #**  **Criteria** | **NST** | | | **ST** | | | | |
| --- | --- | --- | --- | --- | --- | --- | --- | --- |
|  | 1 | 2 | 3 | 1 | 2 | 3 | 4 | 5 |
| **Lactate <2.5 mmol/L** | Yes | Yes | Yes | Yes | Yes | No | Yes | No |
| **Bile production** | Yes | Yes | No | Yes | Yes | Yes | Yes | Yes |
| **Glucose metabolism** | No | Yes | Yes | No | Yes | No | Yes | No |
| **Maintain pH >7.30** | Yes | Yes | Yes | No | No | No | No | No |
| **Stable hemodynamic** | Yes | Yes | Yes | Yes | Yes | Yes | Yes | No |
| **HA flow >150 mL/min** | 265 | 250 | 277 | 217 | 248 | 339 | 563 | 230 |
| **PV flow >500 mL/min** | 1000 | 1200 | 1430 | 1620 | 2170 | 2370 | 1450 | 1240 |
| **Viability** | Yes | Yes | Yes | Yes | Yes | Yes | Yes | No |

^Based on viability criteria reported by Laing et al. (reference 35). A perfused liver is deemed viable if it meets 2 or more criteria. NST, non-steatotic liver; ST, steatotic liver; HA, hepatic artery; PV, portal vein.^
